# Controllable Tunneling Triboelectrification of Two-Dimensional Chemical Vapor Deposited MoS_2_

**DOI:** 10.1038/s41598-018-36830-1

**Published:** 2019-01-23

**Authors:** He Wang, Chung-Che Huang, Tomas Polcar

**Affiliations:** 1 National Centre for Advanced Tribology, Faculty of Engineering and the Environment, University of Southampton, Southampton, SO17 1BJ UK; 2 Optoelectronics Research Centre, University of Southampton, Southampton, SO17 1BJ UK; 3 Department of Control Engineering, Faculty of Electrical Engineering, Czech Technical University in Prague, Technicka 2, 16627 Prague 6, Czech Republic

## Abstract

Tunneling triboelectrification of chemical vapor deposited monolayer MoS_2_ has been characterized at nanoscale with contact-mode atomic force microscopy (AFM) and Kelvin force microscopy (KFM). Although charges can be trapped on insulators like SiO_2_ by conventional triboelectrification, triboelectric charges tunneling through MoS_2_ and localized at the underlying substrate exhibit more than two orders of magnitude longer lifetime. Their polarity and density can be modified by triboelectric process with various bias voltages applied to Pt-coated AFM tips, and the saturated density is almost 30 times higher than the reported result of SiO_2_. Thus, the controllable tunneling triboelectric properties of MoS_2_ on insulating substrates can provide guidance to build a new class of two-dimensional (2D) MoS_2_-based nanoelectronic devices.

## Introduction

Triboelectrification, a contact-induced electrification when a material becomes charged after brought into contact with another one through friction, is a common phenomenon in our daily life^1,2^. For example, charges formed on airplane by air friction when flying can interfere with the radio frequency communication or even cause the plane to be hit by lightning. Although triboelectrification has been known for centuries, it is only until recently that this phenomenon has been utilized as energy convertors and self-powered mechanical sensors^3–7^, particularly electronics like transistors, whose gates shapes, positions and on/off states can be triboelectrically modified on demand^8^.

To date, research about the triboelectrification of insulators^9–12^ as well as cutting-edge zero-gap graphene^8,13^ has been extensively reported, but for other non-zero gap semiconductors like transition metal dichalcogenides, which are good candidates for transistors, there are still a limited number of publications.

As a member of transition metal dichalcogenides family, MoS_2_ shares many attributes of graphene but its non-zero bandgap offers opportunities unattainable for graphene, so it has been widely investigated in the past decade. According to its natural abundance and distinctive optical^14,15^, electrical^16,17^, and mechanical^18,19^ properties, MoS_2_ has become the driving force behind a series of applications, including optoelectronics^20,21^, sensors^22,23^, electronics^24,25^, and energy storage devices^26,27^. For instance, monolayer MoS_2_-based field effect transistors exhibits a remarkably high on/off ratio at room temperature. Gas sensors realized by few-layered MoS_2_ show high sensitivity to the detection of nitrogen monoxide. In the meantime, chemically exfoliated nanosheets of MoS_2_ have been utilized to fabricate batteries and capacitors. However, studies concerning the triboelectric properties of MoS_2_ are poorly reported^28^. As 2D materials exhibit high charge density^29^, it is of great essence to investigate the triboelectric properties of single-layer MoS_2_, so that the results can lay the foundation for its applications in triboelectric nanodevices.

In this work, high-quality 2D MoS_2_ films were synthesized by chemical vapor deposition (CVD) method. The concept of tunneling triboelectrification was introduced to define the tunneling of conventionally friction-induced charges (between AFM and MoS_2_) through the 2D MoS_2_ and their storage on the underlying insulating substrates. The diffusion, manipulation and saturation of tunneling triboelectric charges were investigated with the aid of AFM working in contact and KFM modes. Taking advantage of the capability of accurately controlled triboelectric process, surface charges can be patterned with various shapes and areas in nano-range. And importantly, their polarity and density can also be dynamically altered over time by applying various bias voltages to the conducting AFM tip during rubbing process. Given that the AFM system can be completely integrated on a single chip^30–32^, the realization of time-variant electronic systems and devices based on MoS_2_ is within reach.

## Materials and Methods

### Synthesis of MoS_2_ and transfer method

All the MoS_2_ samples were grown on Si substrate coated with 300 nm SiO_2_. As displayed in Fig. S1, Supporting Information, the atmospheric pressure CVD reaction was carried out in a 3 cm diameter quartz tube, which was surrounded by a furnace and a heating tape. One end of the tube was linked to the gas inlet, through which gases such as nitrogen (N_2_), hydrogen (H_2_), argon (Ar), and hydrogen disulfide (H_2_S) can be introduced, the other end was connected to the exhaust extract component. Before the reaction, boats and substrates were cleaned with acetone, isopropanol and deionized water, and then blow-dried by the nitrogen gun. Subsequently, SiO_2_/Si substrates were placed on a boat containing 10 mg MoO_3_ (Alfa Aescar 99.9995%), and the boat was then pushed into the tube center. After that, another boat containing 100 mg sulfur (Alfa Aescar 99.9995%) was loaded at the upstream, whose temperature was separately controlled by the heating tape instead of the furnace. Later on, 300 standard cubic centimeters per minute (sccm) Ar gas (BOC, 99.999% pure with additional purifications) was introduced to purge the tube for 10 minutes. Afterwards, Ar flow was decreased to 30 sccm, and the furnace temperature was programmed to heat substrates and vaporize MoO_3_ to 550 °C at 25 °C/min ramping rate. After holding this temperature for 5 minutes, it was set to 700 °C at 10 °C/min ramping rate, and sulfur was heated to 200 °C so its vapor could be transported to the tube center, where CVD reaction occurred and MoS_2_ was deposited. After 15 minutes deposition at 700 °C, the heating system was turned off to cool down naturally. The temperature profile of the whole process is plotted in Fig. S2.

The CVD-grown MoS_2_ on SiO_2_/Si was spin-coated with polystyrene (PS, Sigma-Aldrich) at the speed of 1000 rpm for 50 s, and then cured on a hot plate at 90 °C for 15 min. The coated sample was subsequently covered with thermal release tape (from Nitto) and floated in deionized water. Once the MoS_2_/PS/tape stack detached from the substrate, it was transferred to the target substrates (gold, sapphire and polyimide), and the tape was peeled off when dry. The transferred sample was then immersed in CHCl_3_ solvent until the PS film was fully dissolved. Finally, solvent residues of the successfully transferred MoS_2_ samples were rinsed off by deionized water.

### Characterization of synthesized MoS_2_

Scanning electrons microscopy (SEM, JEOL JSM-6500F) and AFM (Scanning Probe Microscopy 5500, Agilent Technologies) were used to investigate morphology of the as-deposited MoS_2_ sample. Raman spectroscopy (InVia Raman Spectrometer, with 532 nm excitation laser) and X-ray photoelectron spectroscopy (XPS, Thermo Scientific Theta Probe XPS System MC03) were utilized to characterize the vibrational modes and elemental composition of as-fabricated nanomaterial.

### Initiation and measurement of triboelectric charges

Contact-mode AFM was performed under a 25 nN applied force at a 1 Hz scan rate to generate triboelectric charges on MoS_2_ with a Pt-coated conductive probe (OMCL-AC240TM-R3 from Olympus). Different bias voltages from −10 to 10 V were applied on the tip to induce a friction pattern on the sample in some experiments. The surface charge was then characterized by KFM mode at ambient environment.

## Results and Discussion

**Figure 1:**
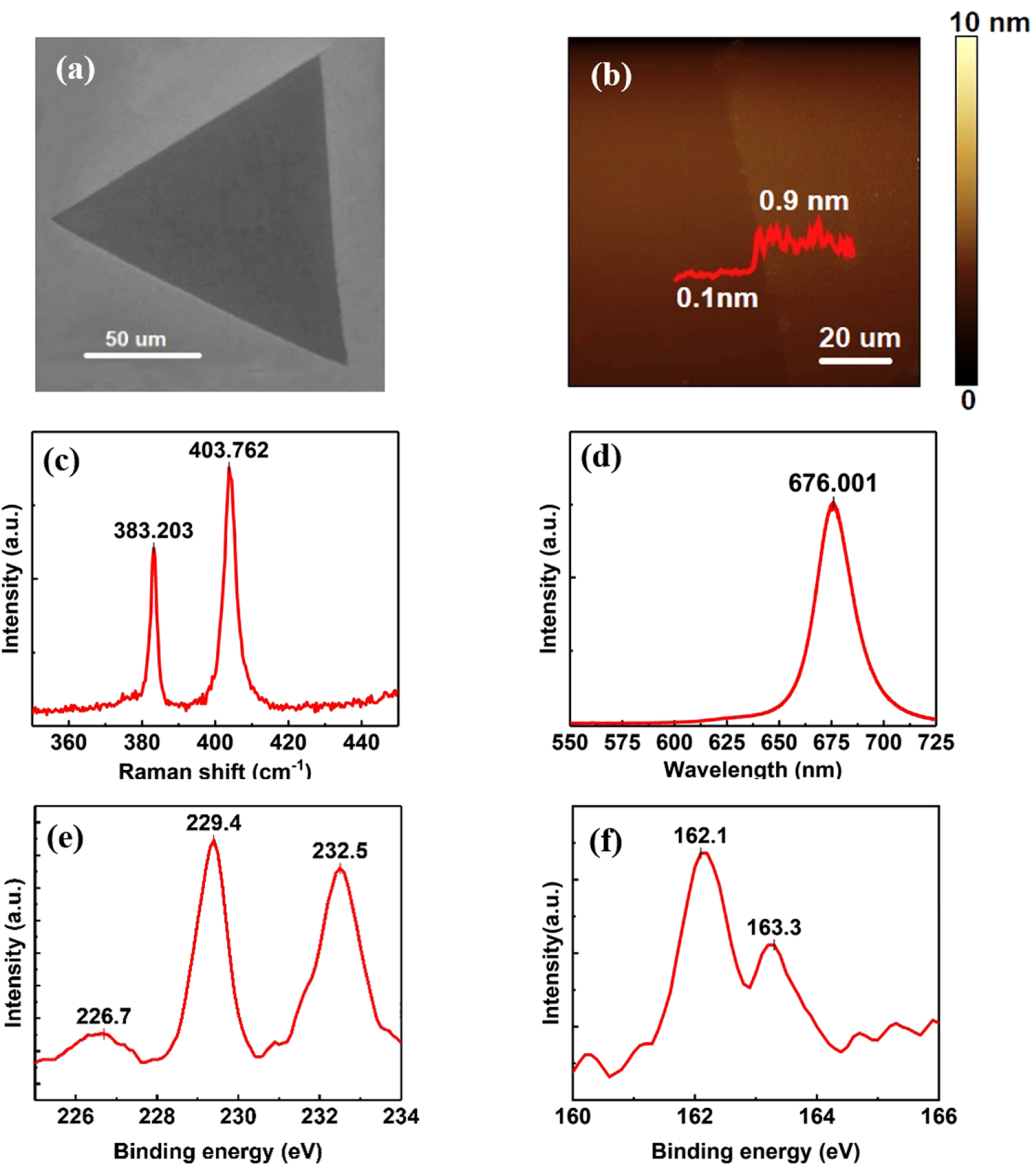
Characterization of MoS_2_ on SiO_2_/Si substrate by CVD method. (**a**) SEM image. (**b**) Surface topography by AFM in tapping mode. (**c**) Raman spectrum and (**d**) PL spectrum excited by 532 nm laser. (**e**) Mo 3d, S 2s orbitals and (**f**) S 2p orbitals demonstrated by XPS spectra.

Figure 1a displays the SEM image of MoS_2_ synthesized on SiO_2_/Si substrate by CVD method. It is noteworthy that the fabricated MoS_2_ sample exhibits a triangular shape, and the lateral size can reach up to ~120 µm, whose limitation may result from the lattice mismatch between the MoS_2_ and SiO_2_/Si substrate. As evident from the surface topography measured by tapping-mode AFM in Fig. 1b, the step height of monolayer MoS_2_ from substrate is ~0.8 nm, which is consistent with the reported thickness of mechanically exfoliated MoS_2_ monolayers^33–38^. Considering the 0.3 nm thickness of S-Mo-S sandwich structure of MoS_2_ single-layer^39–42^, the air gap between the MoS_2_ monolayer and SiO_2_ substrate is estimated as 0.5 nm.

As can be seen in Fig. 1c, there are two characteristic peaks in the Raman spectrum: 383.2 cm^−1^ peak (E_2g_ mode) and 403.8 cm^−1^ peak (A_1g_ mode)^43,44^. The frequency difference between E_2g_ and A_1g_ modes is calculated to be 20.6 cm^−1^ in this study, corresponding to monolayer MoS_2_ ^23,45,46^. Besides, the obvious emission peak at 676 nm in the photoluminescence (PL) spectrum (Fig. 1d) is also indicative of the single-layer nature of the as-fabricated MoS_2_ ^47,48^.

XPS was performed with Al K*α* source to determine the chemical composition of the film. It can be noted from Fig. 1e that the Mo 3d peaks at 229.4 and 232.5 eV are the 3d_3/2_ and 3d_5/2_ orbitals of MoS_2_, respectively; the peak at 226.7 eV belongs to the S 2s orbital. Figure 1f shows the S 2p peaks at 162.1 and 163.3 eV corresponding to the 2p_1/2_ and 2p_3/2_ doublets of S^49^. All these binding energies are in agreement with the reported values for MoS_2_ crystal^50,51^, and the atomic ratio of Mo and S is close to the stoichiometric 1:2. In addition, the XPS spectrum of C 1s orbital is provided in Fig. S3. The peak at 284.6 eV corresponds to the C-C bond, which is expected due to the utilization of carbon tapes for fixing the sample. However, there is no obvious C-S bond (in the 285–287.5 eV range) found in the spectrum^52^, indicating that there is no significant chemisorption of C. Although physisorption could happen, contact-mode AFM would remove adsorbed molecules at the side of tested areas or even wear mateirals^53^. In the meantime, the surface topographic images in Fig. S5 show no accumulation of materials at the side, so it is not expected to have significant contamination on the sample surface.

**Figure 2:**
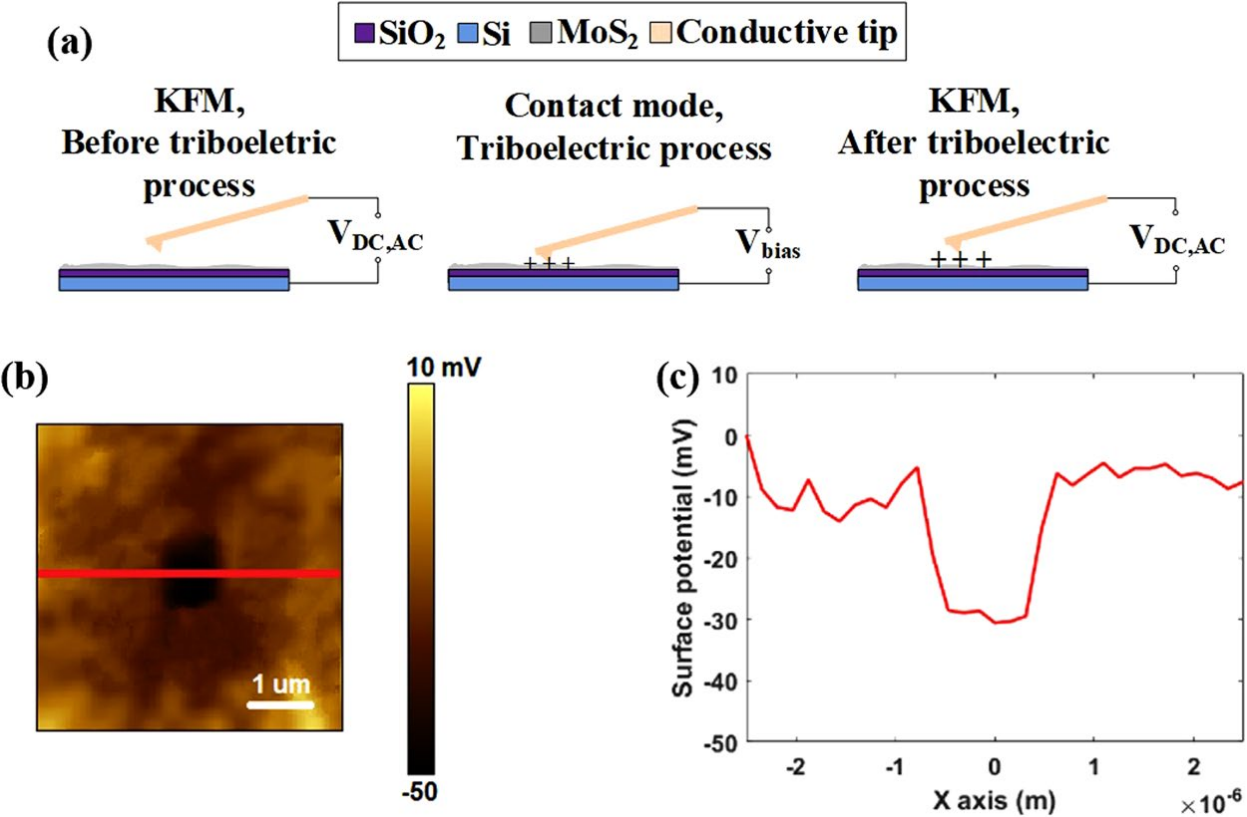
Schematic illustration of triboelectric experiments based on AFM and KFM. (**a**) Surface potential characterizations in KFM mode before and after charge generation by triboelectrification with contact-mode AFM. (**b**) Surface potential image and (**c**) cross-section profile of the potential distribution along the red line in b after rubbing process.

The surface potential before and after triboelectrification was monitored by combining contact-mode AFM and KFM, as illustrated in Fig. 2a. Contact-mode AFM was operated to rub a 1 × 1 µm^2^ square area of the MoS_2_ sample by a conducting tip under a normal force of 25 nN, with the underlying Si grounded. KFM mode was carried out to image the tip-sample contact potential difference of a larger area (5 × 5 µm^2^) with the rubbed area centered. As evident in Fig. S4, the MoS_2_ surface is equipotential except for random fluctuations (probably induced by the absorbed charges from air or contamination) before triboelectrification. After rubbing, the topographic change is undetectable (Fig. S5) but the distinction in the surface potential image is obvious between the rubbed and unrubbed regions (Fig. 2b). The cross-section profile in Fig. 2c demonstrates that the surface potential of the rubbed section in the center is ~20 mV lower than that of the other region. Generally, contact potential difference is determined by two elements: electrostatic potential difference and effective work functions of two materials. The former one is dependent on the surface charge and applied bias, and the latter one is governed by the surface properties of materials^54^. Since the effective work function is almost identical across the sample surface, the drop of surface potential in the rubbed region is induced by the triboelectric charging. In this case, a fraction of negative charges were transferred to the sample surface via triboelectrification, which lowered the surface potential of the central region.

**Figure 3:**
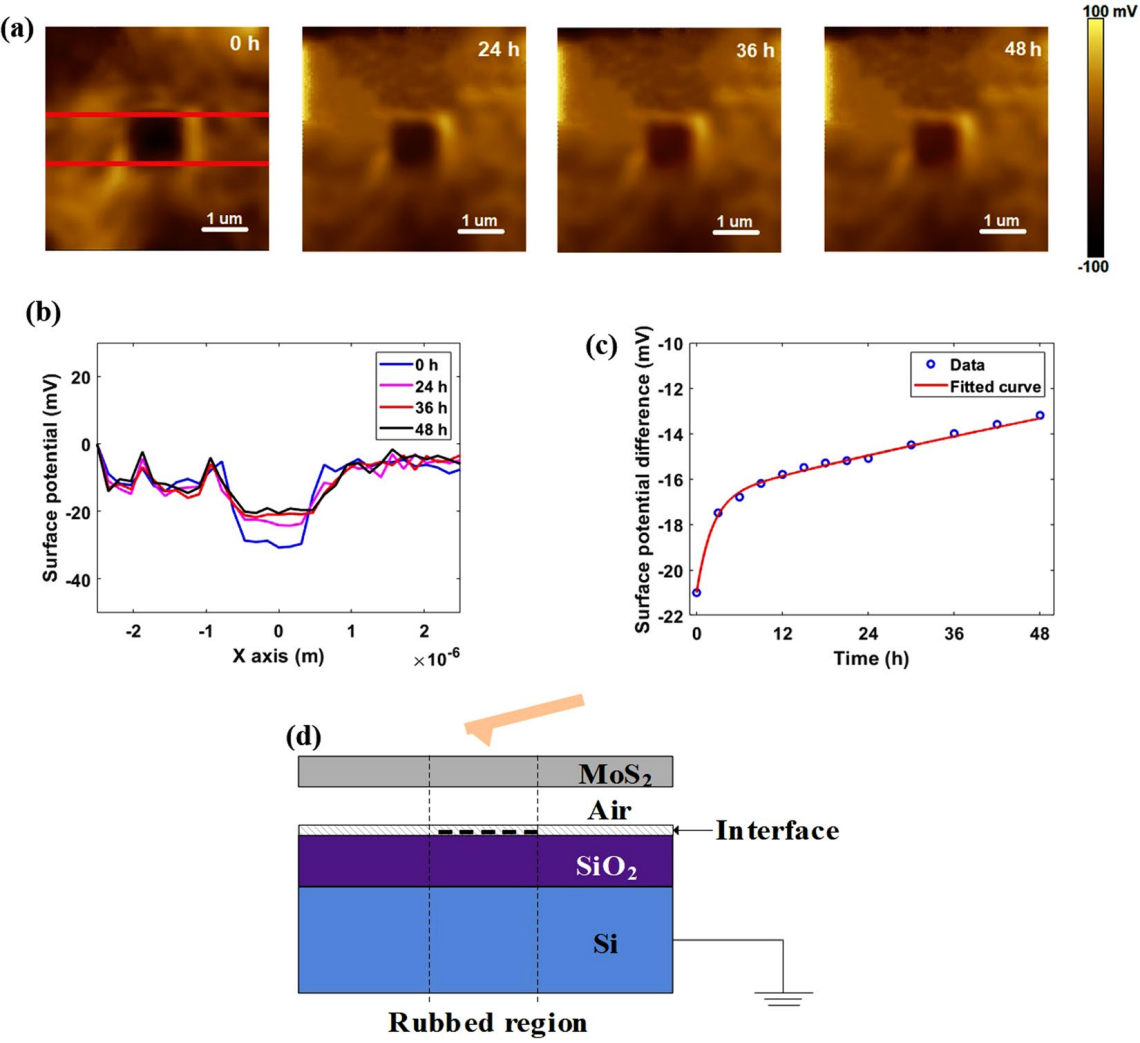
Diffusion of triboelectric charges over time. (**a**) Surface potential images after 0, 24, 36 and 48 hours. (**b**) Cross-section profiles between the lines in a. (**c**) Surface potential difference as a function of time and its fitted curve. (**d**) Schematic diagram for tunneling triboelectrification.

Figure 3a,b show the surface potential maps and their cross-section profiles (corresponding to the stripy region confined by the two red lines in Fig. 3a, the surface potentials were averaged to reduce the influence of random fluctuations) after triboelectrification for up to 48 hours. It is noteworthy that the potential difference remains detectable even after two days. As charges can be kept for a long time only on insulating materials, such variation cannot originate from the charges on MoS_2_. Here we suggest that some triboelectric charges generated by triboelectrification tunnel through the single-layer MoS_2_ and localize on the insulator underneath.

Additional experiments have also been conducted with other substrates, consisting of conductive gold as well as insulators like sapphire and polyimide (Figs S6–S8). Similar to the case of SiO_2_, triboelectric charges are stored at the air-insulator interface after tunneling through MoS_2_ for sapphire and polyimide. However, there is no detectable localized charges in the rubbed section with gold as the substrate. As the surface potential of the whole area is slightly lower after triboelectrification, it is suggested that the transferred charges spread across the whole MoS_2_ or gold substrate due to the excellent conductivity of gold.

The surface potential differences of the rubbed versus unrubbed regions on sapphire and polyimide decreased by ~10 mV after 12 hours, but in the case of SiO_2_ substrate, ~5 mV decrease was observed (Fig. 3c), which indicates a better preservation of tunneling triboelectric charges. Therefore, we used MoS_2_ monolayers on SiO_2_ substrate for further investigation.

As charges cannot diffuse through insulators like air and SiO_2_, these tunneling triboelectric charges are localized on the air-SiO_2_ interface, and attract opposite charges from MoS_2_ and/or Si underneath SiO_2_, as the schematic diagram shown in Fig. 3d. Considering that the thickness of air (0.5 nm) is far smaller than that of the SiO_2_ (300 nm), the capacitance of air is even larger, so almost all the opposite charges are donated by MoS_2_ and accumulate across the air gap, while the stored charges on SiO_2_ layer are almost unvaried. Consequently, the measured surface potential difference across MoS_2_ is almost identical to the voltage change through the air gap.

After triboelectrification, no current flow will be detected in MoS_2_ once the equilibrium is reached, and leakage currents will be the only reason for the discharge of both air and SiO_2_, so two time constants will be developed. Figure 3c shows the relationship between surface potential difference (Δ*V*) with time and its fitted function is depicted as follows:

1 $${\rm{\Delta }}V=-\,4.2\times {e}^{\frac{-t}{{\tau }_{short}}}-16.8\times {e}^{\frac{-t}{{\tau }_{long}}}$$

As evident, apart from a small term with a shorter time constant (*τ*_*short*_ ~ 2.17 h), the other term owns a larger initial amplitude and a longer time constant (*τ*_*long*_ ~ 207.8 h), which is significantly longer than the ~1 h decay time of triboelectric charges on SiO_2_ with identical thickness of 300 nm^55^. The dominant term is attributed to the small-area air gap: the initial amplitude is greater since most of the charges are attracted from MoS_2_ and trapped across the air gap; the longer time constant originates from the great insulating nature of air and the protection of MoS_2_ film.

**Figure 4:**
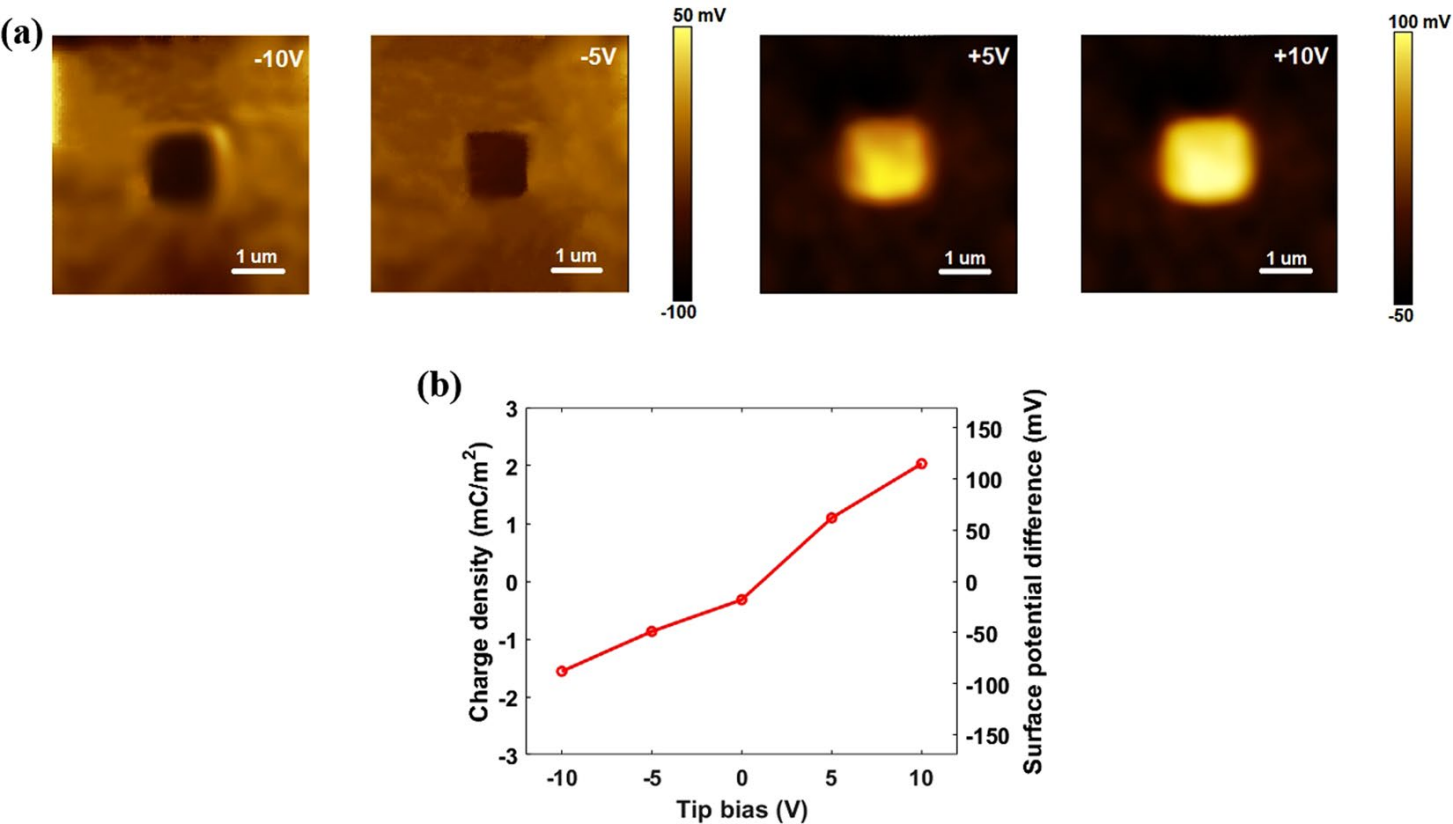
Manipulation of polarity and density of tunneling triboelectric charges. (**a**) Surface potential images after rubbing the central region with −10, −5, +5 and +10 V biases. (**b**) Charge density and surface potential difference the as a function of tip bias.

The polarity and density of tunneling triboelectric charges on MoS_2_ can be controlled by applying different bias voltages to the conducting Pt-coated AFM tip during triboelectric process. As can be seen from the surface potential images after rubbing with biases ranging from −10 to +10 V in Fig. 4a, positive charges are trapped at the interface and electrons are attracted in the central region of MoS_2_ at positive bias voltages, resulting in an n-type MoS_2_; in contrast, negative charges are injected across the air gap and holes are attracted in the rubbed section with negative biases, so a p-type MoS_2_ is formed. As surface potential difference between the intact and rubbed sections is almost identical to the voltage drop through the air gap, charge density σ of tunneling triboelectrification can be calculated based on capacitor model as:

2 $$\sigma =\frac{{\rm{\Delta }}V{\varepsilon }_{0}{\varepsilon }_{Air}}{{t}_{Air}}$$

where Δ*V* is the voltage drop through the air gap, *ε*_0_ is the vacuum dielectric constant, *ε*_*Air*_ and *t*_*Air*_ are the relative dielectric constant and thickness of air gap, respectively.

As evident from the surface potential differences and corresponding charge densities in Fig. 4b, the surface potential difference increases with the applied voltage non-linearly, which results from the different energy state densities within the bandgap.

**Figure 5:**
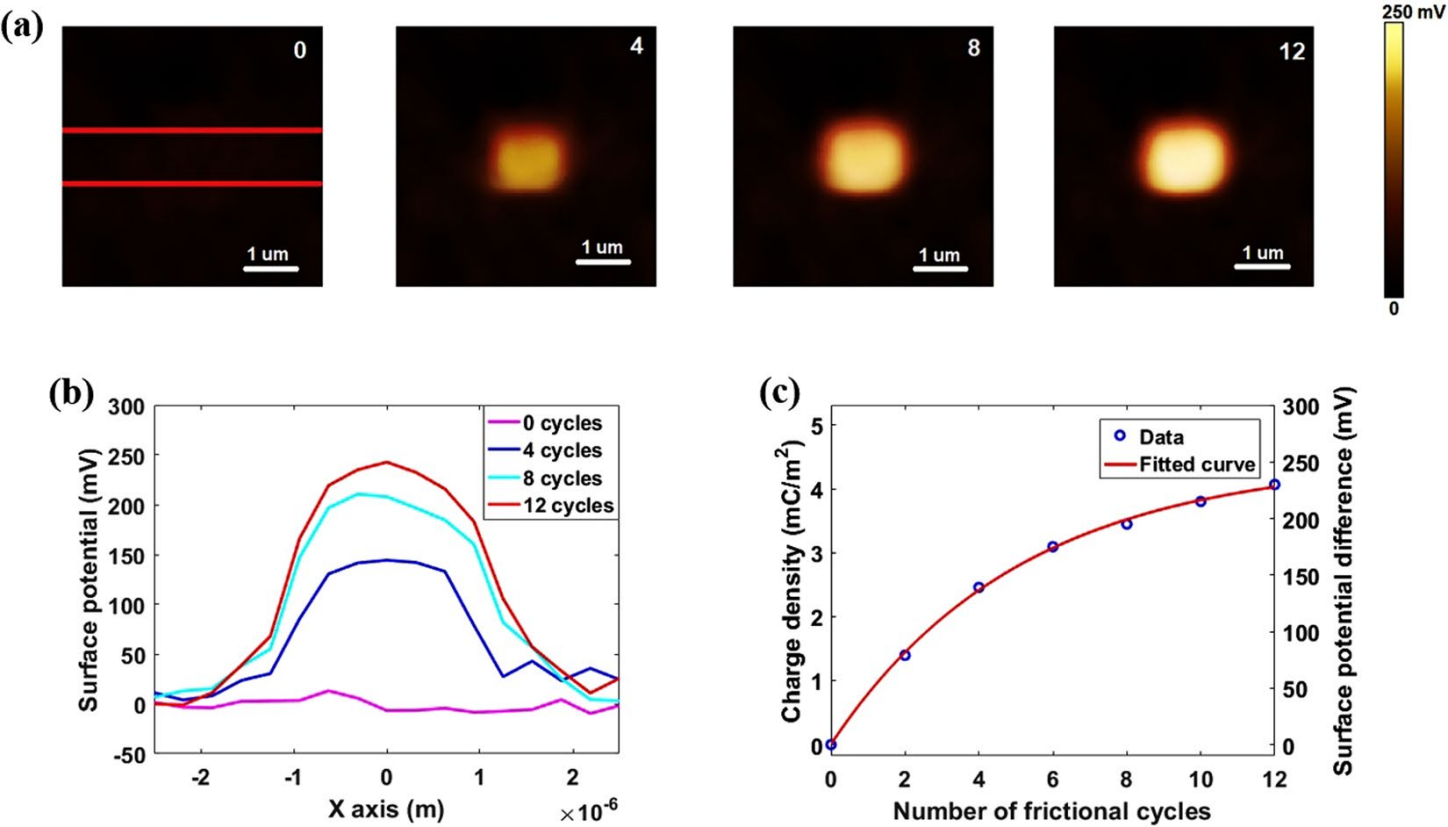
Accumulation of triboelectric charges with rubbing cycles increased. (**a**) Surface potential images after rubbing with 0, 4, 8 and 12 cycles. (**b**) Cross-section profiles between the lines in a. (**c**) Charge density and surface potential difference as a function of frictional cycles number.

Now we explore the ability of AFM system to provide multiple scans of selected area in order to increase the accumulated charge. The as-deposited MoS_2_ sample was rubbed for multiple cycles with a constant contact force of 25 nN and bias voltage of +10 V; corresponding surface potential images are shown in Fig. 5a. As the whole measurement was completed within 30 minutes, the influence of charge diffusion can be neglected considering the good preservation of tunneling triboelectric charges. Figure 5b displays the averaged surface potential differences and relevant charge densities within these 12 cycles, and an obvious accumulation and saturation trend for the triboelectric charge can be seen.

In previous reports^56,57^, the mechanism of triboelectric process can be described by the assumption of effective work function: the amount of transferred charges in each cycle is linked with the difference of effective work functions between the tip and sample. The difference will reduce with the charge accumulation process until a saturation is reached. A phenomenological model can be used to fit the experimental data as illustrated in equation 3:

3 $$\frac{d\sigma }{dn}={{\rm{kV}}}_{c}-p{{\rm{V}}}_{e}\sigma $$

where *σ* and *n* are the surface charge density and the number of rubbing cycles, respectively, k is the charge efficiency coefficient, V_*c*_ is representative of the work function difference between the tip and sample, *p* is the charge impedance coefficient, and V_*e*_ is the charge-induced potential on measured surface.

Considering the boundary conditions for equation 3, the following relationship can be obtained:

4 $$\sigma ={\sigma }_{0}\,\exp (-\frac{n}{{n}_{0}})+{\sigma }_{\infty }[1-\exp (-\frac{n}{{n}_{0}})]$$

where *σ*_0_ and *σ*_*∞*_ are the surface charge densities at *n* = 0 and *n* = ∞, respectively. *n*_0_ is a constant indicating the speed of charge saturation.

By fitting the experimental data for different rubbing cycles (Fig. 5c), all the parameters can be extracted: *σ*_0_ = (8.01 ± 0.01) µC/m^2^, *σ*_∞_ = (4.46 ± 0.01) mC/m^2^, *n*_0_ = (5.14 ± 0.01). Compared with the recently published results, the saturated tunneling triboelectric charge density of MoS_2_ (~4.46 mC/m^2^) is significantly higher than that of SiO_2_ (~150 µC/m^2^)^55^, and it can lay the foundation for triboelectric applications^28^. As for the enhancement of charge density, it mainly results from the tunneling of triboelectric charges, which are kept at the interlayer between the MoS_2_ monolayer and the underlying SiO_2_/Si substrate. Under this circumstance, the MoS_2_ layer as well as the thin air gap protect these charges from being neutralized by charges or ions in the air, thereby improving the charge density compared with the case of SiO_2_.

## Conclusions

To conclude, nanoscale tunneling triboelectrification of chemical vapor deposited MoS_2_ film was demonstrated by linking contact-mode AFM with KFM techniques at ambient environment. The charge transfer and the following diffusion process, as well as the accumulation from multi-friction effect were observed systematically. We show that the tunneling triboelectric charges can be preserved for an impressively long time, which is 100 times longer than that of the charges induced by conventional triboelectrification. Moreover, both positive and negative tunneling triboelectric charges can be induced without changing the surface topography by applying different bias voltages to the conductive tips via frictional process, and their density is controllable. Besides, a saturation of localized charges can be reached through multi-friction. These unique properties can pave the way for the development of novel 2D MoS_2_-based nanodevices, especially the triboelectrically controlled transistors, which can reduce the energy consumption to a large extent compared with the currently used voltage-controlled one.

## Electronic supplementary material

Supplementary Information

## References

[1] Horn RG, Smith DT (1992). Dissimilar. Materials..

[2] Horn RG, Smith DT, Grabbe A (1993). Contact electrification induced by monolayer modification of a surface and relation to acid–base interactions. Nature.

[3] Wang ZL (2013). Triboelectric nanogenerators as new energy technology for self-powered systems and as active mechanical and chemical sensors. ACS Nano.

[4] Zhu G (2012). Triboelectric-generator-driven pulse electrodeposition for micropatterning. Nano Lett..

[5] Fan FR, Tian ZQ, Lin Wang Z (2012). Flexible triboelectric generator. Nano Energy.

[6] Wang S, Lin L, Wang ZL (2012). Nanoscale triboelectric-effect-enabled energy conversion for sustainably powering portable electronics. Nano Lett..

[7] Zhu G (2013). Toward large-scale energy harvesting by a nanoparticle-enhanced triboelectric nanogenerator. Nano Lett..

[8] Kim S (2017). Rewritable ghost floating gates by tunnelling triboelectrification for two-dimensional electronics. Nat. Commun..

[9] Li HY (2015). Significant Enhancement of Triboelectric Charge Density by Fluorinated Surface Modification in Nanoscale for Converting Mechanical Energy. Adv. Funct. Mater..

[10] Zhu H (2016). Triboelectric Nanogenerators Based on Melamine and Self-Powered High-Sensitive Sensors for Melamine Detection. Adv. Funct. Mater..

[11] Wang S (2016). Molecular surface functionalization to enhance the power output of triboelectric nanogenerators. J. Mater. Chem. A.

[12] Kim, J. H., Yun, B. K., Jung, J. H. & Park, J. Y. Enhanced triboelectrification of the polydimethylsiloxane surface by ultraviolet irradiation. *Appl. Phys. Lett*. **108** (2016).

[13] Bdikin I (2017). Charge injection in large area multilayer graphene by ambient Kelvin probe force microscopy. Appl. Mater. Today.

[14] Wi, S. *et al*. Photovoltaic response in pristine WSe2 layers modulated by metal-induced surface-charge-transfer doping. *Appl. Phys. Lett*. **107** (2015).

[15] Zeng H, Dai J, Yao W, Xiao D, Cui X (2012). Valley polarization in MoS_2_ monolayers by optical pumping. Nat. Nanotechnol..

[16] Radisavljevic B, Radenovic A, Brivio J, Giacometti V, Kis A (2011). Single-layer MoS_2_ transistors. Nat. Nanotechnol..

[17] Lopez-Sanchez O, Lembke D, Kayci M, Radenovic A, Kis A (2013). Ultrasensitive photodetectors based on monolayer MoS_2_. Nat. Nanotechnol..

[18] Bertolazzi S, Brivio J, Kis A (2011). Stretching and breaking of ultrathin MoS_2_. ACS Nano.

[19] Castellanos-Gomez A (2012). Elastic Properties of Freely Suspended MoS_2_ Nanosheets. Adv. Mater..

[20] Lee HS (2012). MoS_2_ nanosheet phototransistors with thickness-modulated optical energy gap. Nano Lett..

[21] Han SA, Bhatia R, Kim S-W (2015). Synthesis, properties and potential applications of two-dimensional transition metal dichalcogenides. Nano Converg..

[22] Wang Z (2012). Comparative studies on single-layer reduced graphene oxide films obtained by electrochemical reduction and hydrazine vapor reduction. Nanoscale Res. Lett..

[23] Li H (2012). Fabrication of single- and multilayer MoS_2_ film-based field-effect transistors for sensing NO at room temperature. Small.

[24] Wang H (2012). Integrated circuits based on bilayer MoS_2_ transistors. Nano Lett..

[25] Zhang Y, Ye J, Matsuhashi Y, Iwasa Y (2012). Ambipolar MoS_2_ thin flake transistors. Nano Lett..

[26] Acerce M, Voiry D, Chhowalla M (2015). Metallic 1T phase MoS_2_ nanosheets as supercapacitor electrode materials. Nat. Nanotechnol..

[27] Chang K, Chen W (2011). L-Cysteine-assisted synthesis of layered MoS_2_/graphene composites with excellent electrochemical performances for lithium ion batteries. ACS Nano.

[28] Wu, C. *et al*. Enhanced Triboelectric Nanogenerators Based on MoS_2_ Monolayer Nanocomposites Acting as Electron-Acceptor Layers. *ACS Nano* acsnano. 7b03657, 10.1021/acsnano.7b03657 (2017). 10.1021/acsnano.7b03657 28737887

[29] Müller-Caspary K (2018). Atomic-scale quantification of charge densities in two-dimensional materials. Phys. Rev. B.

[30] Vettiger P (2002). The ‘Millipede’ - Nanotechnology Entering Data Storage. IEEE Trans. Nanotechnol..

[31] Chu LL, Takahata K, Selvaganapathy PR, Gianchandani YB, Shohet JL (2005). A micromachined Kelvin probe with integrated actuator for microfluidic and solid-state applications. J. Microelectromechanical Syst..

[32] Barrettino D (2005). CMOS monolithic mechatronic microsystem for surface imaging and force response studies. in. IEEE Journal of Solid-State Circuits.

[33] Kim Y, Bark H, Ryu GH, Lee Z, Lee C (2016). Wafer-scale monolayer MoS_2_ grown by chemical vapor deposition using a reaction of MoO3 and H2S. J. Physics-Condensed Matter.

[34] Liu H (2016). Role of the carrier gas flow rate in monolayer MoS_2_ growth by modified chemical vapor deposition. Nano Res..

[35] Vangelista, S. *et al*. Ultrafast growth of large-area monolayer MoS_2_ film via gold foil assistant CVD for a highly sensitive photodetector (2017). 10.1088/1361-6528/aa7473 28616939

[36] Senthilkumar V (2014). Direct vapor phase growth process and robust photoluminescence properties of large area MoS_2_ layers. Nano Res..

[37] Yu Y (2013). Controlled scalable synthesis of uniform, high-quality monolayer and few-layer MoS_2_ films. Sci. Rep..

[38] Lee YH (2013). Synthesis and transfer of single-layer transition metal disulfides on diverse surfaces. Nano Lett..

[39] Cammarata A, Polcar T (2015). Tailoring Nanoscale Friction in MX2 Transition Metal Dichalcogenides. Inorg. Chem..

[40] Irving BJ, Nicolini P, Polcar T (2017). On the lubricity of transition metal dichalcogenides: An: ab initio study. Nanoscale.

[41] Nicolini P, Polcar T (2016). A comparison of empirical potentials for sliding simulations of MoS_2_. Comput. Mater. Sci..

[42] Addou R, Colombo L, Wallace RM (2015). Surface Defects on Natural MoS_2_. ACS Appl. Mater. Interfaces.

[43] Li H (2012). From bulk to monolayer MoS_2_: Evolution of Raman scattering. Adv. Funct. Mater..

[44] Zhang, X. *et al*. Raman spectroscopy of shear and layer breathing modes in multilayer MoS_2_. *Phys. Rev. B - Condens. Matter Mater. Phys*. **87** (2013).

[45] Ji Q (2013). Epitaxial monolayer MoS_2_ on mica with novel photoluminescence. Nano Lett..

[46] Liu KK (2012). Growth of large-area and highly crystalline MoS_2_ thin layers on insulating substrates. Nano Lett..

[47] Chen C (2015). Growth of large-area atomically thin MoS_2_ film via ambient pressure chemical vapor deposition. Photonics Res..

[48] Van Der Zande AM (2013). Grains and grain boundaries in highly crystalline monolayer molybdenum disulphide. Nat. Mater..

[49] Papageorgopoulos CA, Jaegermann W (1995). Li intercalation across and along the van der Waals surfaces of MoS_2_ (0001). Surf. Sci..

[50] Altavilla C, Sarno M, Ciambelli P (2011). A novel wet chemistry approach for the synthesis of hybrid 2D free-floating single or multilayer nanosheets of MS2@oleylamine (M = Mo, W). Chem. Mater..

[51] Wong KC (2008). Surface and friction characterization of MoS_2_ and WS2 third body thin films under simulated wheel/rail rolling-sliding contact. Wear.

[52] Bratt, A. & Barron, A. R. XPS of CarbonNanomaterials. *Measurement* 1–16 (2011).

[53] Zekonyte J, Polcar T (2015). Friction Force Microscopy Analysis of Self-Adaptive W-S-C Coatings: Nanoscale Friction and Wear. ACS Appl. Mater. Interfaces.

[54] Melitz W, Shen J, Kummel AC, Lee S (2011). Kelvin probe force microscopy and its application. Surf. Sci. Rep..

[55] Zhou YS (2013). *In situ* quantitative study of nanoscale triboelectrification and patterning. Nano Lett..

[56] Matsusaka S, Maruyama H, Matsuyama T, Ghadiri M (2010). Triboelectric charging of powders: A review. Chem. Eng. Sci..

[57] Williams, M. W. Triboelectric charging of insulating polymers-some new perspectives. *AIP Adv*. **2** (2012).

