# Supplementary material for: Controllable Tunneling Triboelectrification of Two-Dimensional Chemical Vapor Deposited MoS2
Source: Sci Rep. 2019 Jan 23;9:334. doi: 10.1038/s41598-018-36830-1 (PMC6344571; doi:10.1038/s41598-018-36830-1)

Supporting Information

Controllable Tunneling Triboelectrification of Two-dimensional Chemical Vapor Deposited MoS_2

He Wang, Chung-Che Huang, Tomas Polcar

Fig. S1 Schematic illustration for the growth of MoS_2 films on the SiO_2/Si substrates by CVD.

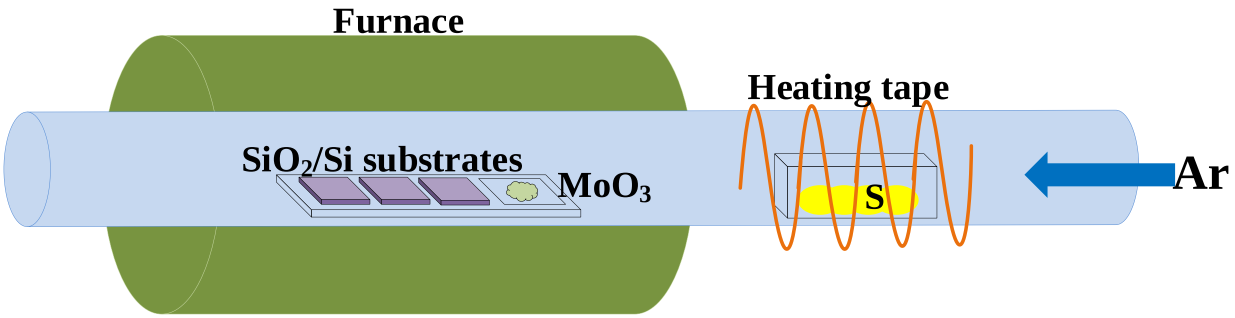

Fig. S2 Temperature profile of MoO_3 (black line) and S (red line).

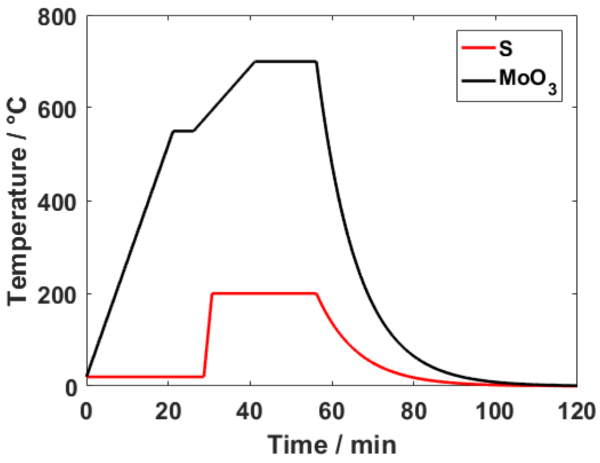

Fig. S3 XPS spectrum of C 1s orbital.

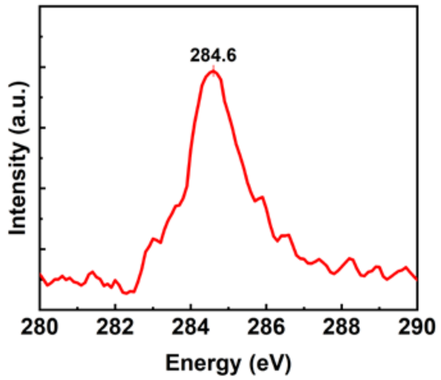

Fig. S4 (a) Surface potential image and (b) cross-section profile of the potential distribution along the red line in (a) before rubbing process (the rubbed area is marked with the blue square).

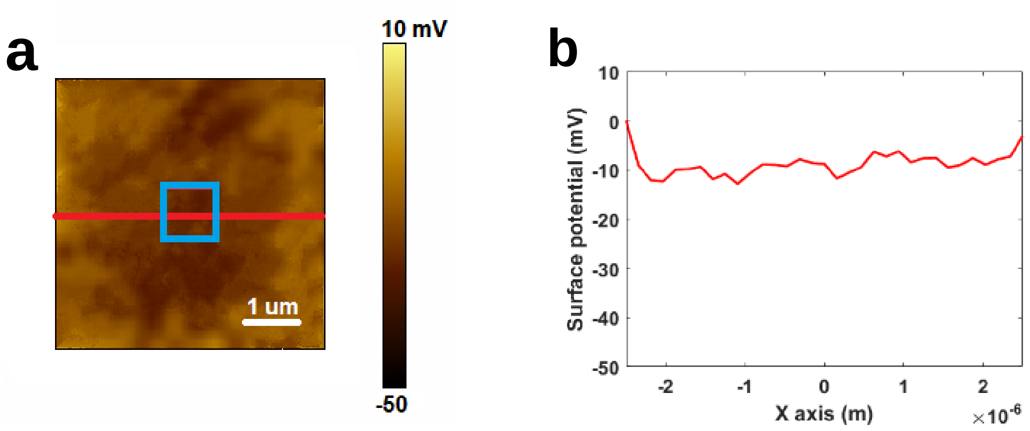

Fig. S5 Surface topography images before and after triboelectric charge generation.

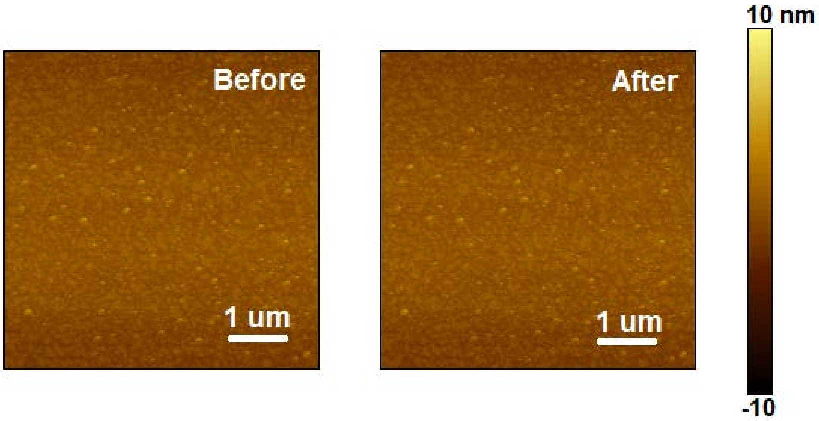

Fig. S6 (a) KFM images of MoS_2 on gold substrate before and after triboelectrification. (b) Cross-section profile along the red line in (a).

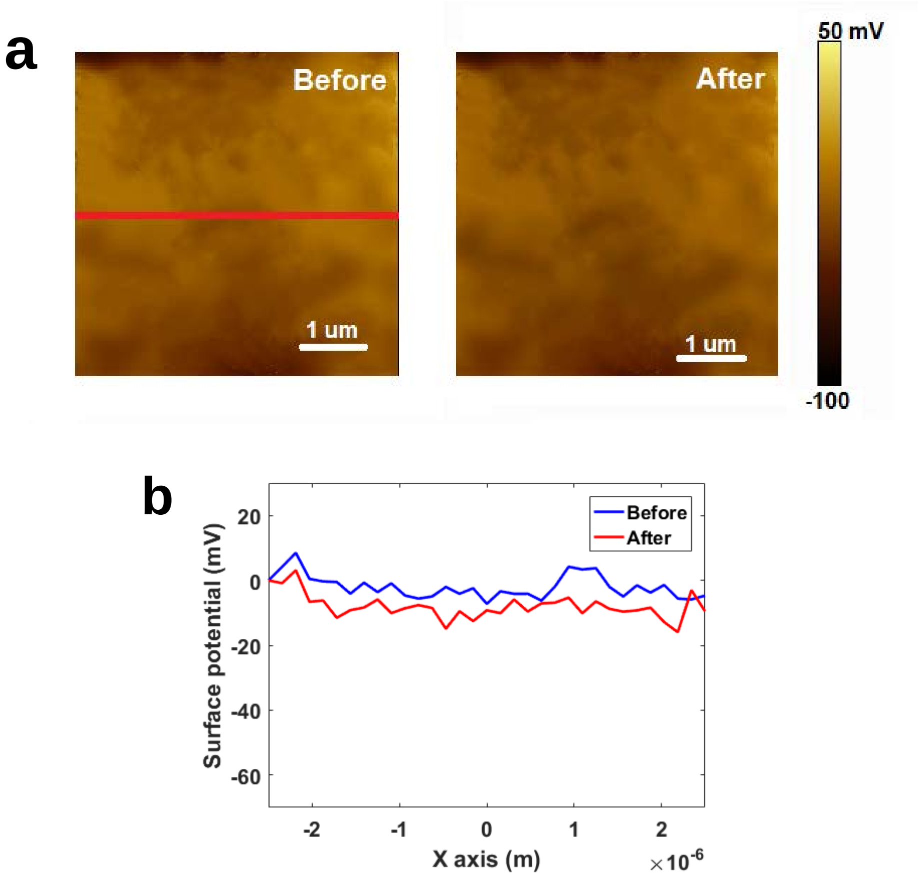

Fig. S7 (a) KFM images of MoS_2 on sapphire substrate after triboelectrification. (b) Cross-section profile along the red line in (a) for 0 and 12 hours.

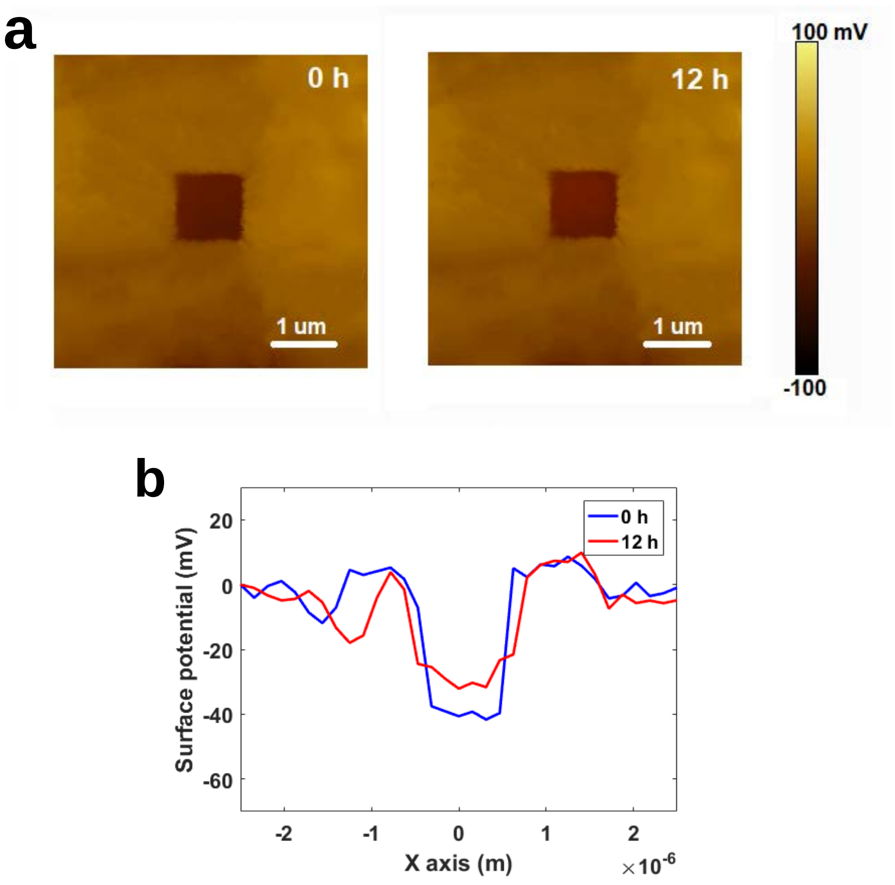

Fig. S8 (a) KFM images of MoS_2 on polyimide substrate after triboelectrification. (b) Cross-section profile along the red line in (a) after 0 and 12 hours.

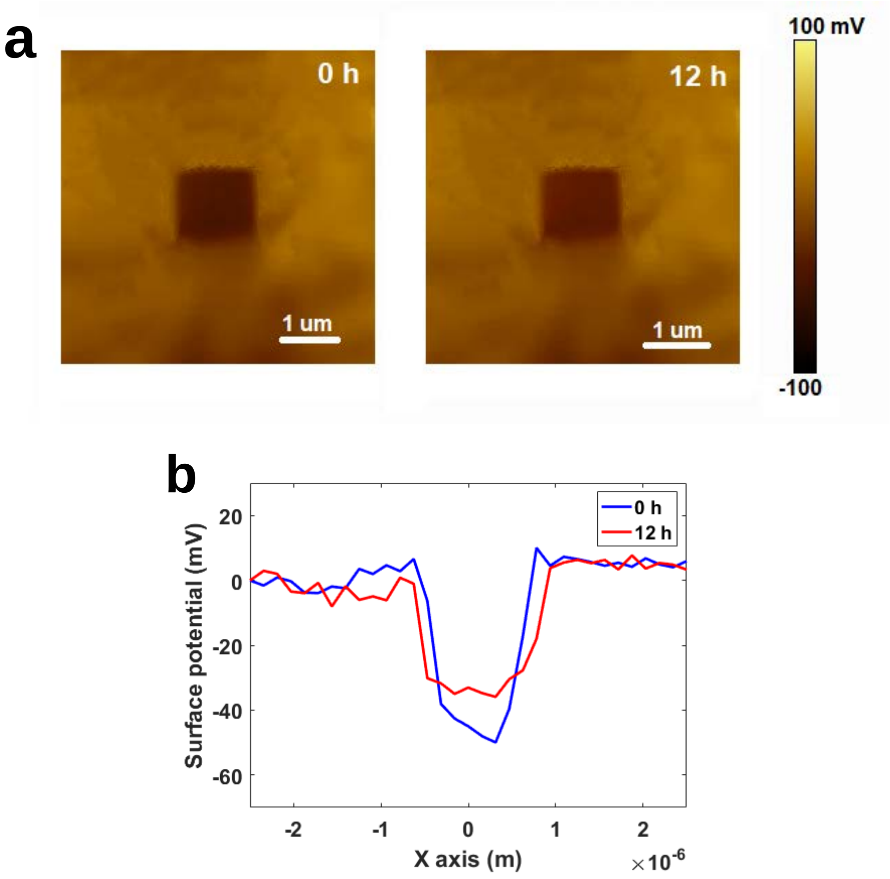

Supplement: Supplementary file 1 — Supplementary Information [file 41598_2018_36830_MOESM1_ESM.pdf]
